# GPN does not release lysosomal Ca^2+^ but evokes Ca^2+^ release from the ER by increasing the cytosolic pH independently of cathepsin C

**DOI:** 10.1242/jcs.223883

**Published:** 2019-02-01

**Authors:** Peace Atakpa, Laura M. van Marrewijk, Michael Apta-Smith, Sumita Chakraborty, Colin W. Taylor

**Affiliations:** Department of Pharmacology, University of Cambridge, Tennis Court Road, Cambridge CB2 1PD, UK

**Keywords:** Ca^2+^ signals, Cathepsin C, Cytosolic pH, Endoplasmic reticulum, GPN, Lysosome

## Abstract

The dipeptide glycyl-l-phenylalanine 2-naphthylamide (GPN) is widely used to perturb lysosomes because its cleavage by the lysosomal enzyme cathepsin C is proposed to rupture lysosomal membranes. We show that GPN evokes a sustained increase in lysosomal pH (pH_ly_), and transient increases in cytosolic pH (pH_cyt_) and Ca^2+^ concentration ([Ca^2+^]_c_). None of these effects require cathepsin C, nor are they accompanied by rupture of lysosomes, but they are mimicked by structurally unrelated weak bases. GPN-evoked increases in [Ca^2+^]_c_ require Ca^2+^ within the endoplasmic reticulum (ER), but they are not mediated by ER Ca^2+^ channels amplifying Ca^2+^ release from lysosomes. GPN increases [Ca^2+^]_c_ by increasing pH_cyt_, which then directly stimulates Ca^2+^ release from the ER. We conclude that physiologically relevant increases in pH_cyt_ stimulate Ca^2+^ release from the ER in a manner that is independent of IP_3_ and ryanodine receptors, and that GPN does not selectively target lysosomes.

## Abstract (precis)

**Highlighted Article:** GPN causes an increase in the cytosolic pH, evoking Ca^2+^ release from the ER that is independent of cathepsin C.

## INTRODUCTION

**Fig. 1.**
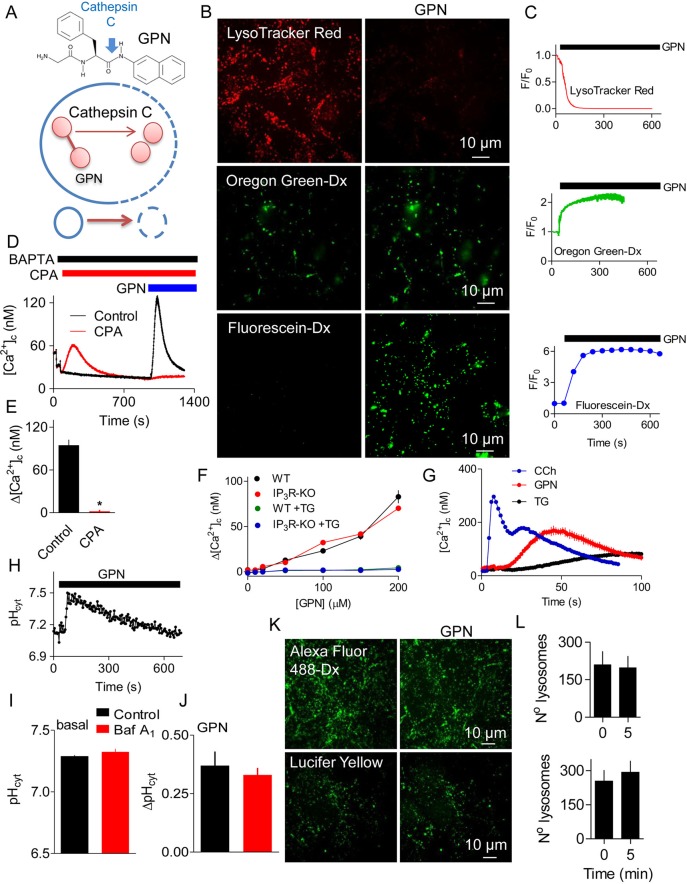
**GPN changes pH and [Ca^2+^]_c_ without rupturing lysosomes.** (A) GPN is proposed to disrupt lysosomes because its cleavage by cathepsin C (blue arrow) causes osmotic lysis. (B) HEK cells loaded with LysoTracker Red, or with dextran conjugates of Oregon Green or fluorescein report an increase in pH_ly_ after addition of GPN (200 µM for 200 s). Increased pH causes fluorescence to decrease for LysoTracker Red and increase for the other indicators. (C) Time courses of GPN-evoked changes in fluorescence (F/F_0_) of the pH_ly_ indicators. Each trace shows mean±s.d. from 3–4 ROIs in a single cell (summarised in Fig. 3F,H). (D) BAPTA (2.5 mM) was added to chelate extracellular Ca^2+^, and cyclopiazonic acid (CPA, 20 µM) to inhibit SERCAs, before the addition of GPN (200 µM) to fluo 8-loaded HEK cells. Results show mean±s.d. for 3 replicates. (E) Summary results (mean±s.e.m., *n*=3) from analyses similar to those in panel D show peak increase in [Ca^2+^]_c_ (Δ[Ca^2+^]_c_) evoked by GPN. *P<0.05, Student's *t*-test. (F) Wild-type HEK cells (WT) or HEK cells without IP_3_Rs (IP_3_R-KO) kept in Ca^2+^-free HBS were either stimulated with 200 µM GPN alone or treated with 1 µM thapsigargin (TG) for 15 min followed by addition of 200 µM GPN. Results (mean±s.e.m., *n*=4, with 3 replicates) show Δ[Ca^2+^]_c_. The green symbols are obscured by the overlying blue symbols. (G) Initial responses of HEK cells to carbachol (CCh, 1 mM), GPN (200 µM) or thapsigargin (TG, 1 µM) in Ca^2+^-free HBS (mean±s.d. of 3 replicates). A summary of these data is shown in Fig. S1C,D. (H) Effect of GPN (200 µM) on pH_cyt_ determined using the pH indicator SNARF-5F in populations of HEK cells. (I,J) Effects of bafilomycin A_1_ (Baf A_1_, 1 µM, 1 h) on basal pH_cyt_ (I) and on the ΔpH_cyt_ evoked by GPN (200 µM, 200 s) (J). Results (mean±s.e.m., *n*=5, each with 3 replicates) show no significant effect of BafA_1_ (Student's *t*-test). (K) Representative confocal images show that GPN (200 µM, 10 min) had no effect on the punctate distribution of endocytosed Lucifer Yellow (*M*_r_ 447) or Alexa Fluor 488-coupled dextran (*M*_r_ ∼10,000). (L) Number of lysosomes identified before (0 min) and 5 min after GPN addition in at least 3 cells per coverslip. Mean±s.e.m., *n*=3 independent coverslips (Alexa Fluor 488-Dx, top graph) and *n*=4 independent coverslips (Lucifer Yellow, bottom graph).

Lysosomes are dynamic, membrane-bound organelles that maintain a low luminal pH (pH_ly_ ∼4.5) (Johnson et al., 2016). More than fifty degradative enzymes within lysosomes allow them to degrade materials imported by endocytosis, and to recycle intracellular materials by autophagy (Luzio et al., 2014; Rubinsztein et al., 2012). Lysosomes coordinate responses to nutrient deprivation through their ability to sense amino acids, and regulate the biogenesis of lysosomes and autophagy proteins (Sabatini, 2017). They also mediate transfer of cholesterol and other lipids between membranes, and they contribute to membrane repair (Thelen and Zoncu, 2017). Lysosomes also sequester Ca^2+^ and express Ca^2+^-permeable channels, notably transient receptor protein mucolipin (TRPML1), two pore channel type 2 (TPC2, encoded by *TPCN2*) and ATP-regulated P_2_X_4_ receptor (P2RX4) (Morgan et al., 2011). These proteins allow Ca^2+^ to regulate lysosomal behaviour and allow lysosomes to contribute to cytosolic Ca^2+^ signalling (López Sanjurjo et al., 2013). There are, however, few pharmacological opportunities to disable lysosomes: inhibition of the lysosomal V-ATPase (with bafilomycin A_1_ or concanamycin A) allows the lysosomal pH gradient to be dissipated (Drose and Altendorf, 1997), and glycyl-l-phenylalanine 2-naphthylamide (l-GPN, hereafter referred to as GPN) is widely used, purportedly to disrupt lysosomal membranes (Fig. 1A).

Many molecules, typically amphipathic weak bases, accumulate within lysosomes because they are sufficiently lipophilic at neutral pH to cross biological membranes, but at low pH_ly_ they are protonated and trapped in the lysosome lumen, where they can become highly concentrated (Nadanaciva et al., 2011). These molecules, which include many drugs in clinical use, are described as ‘lysosomotropic’. Accumulation of lysosomotropic molecules within lysosomes increases pH_ly_, and the high concentrations achieved can perturb lysosomal functions. Sphingosine, for example, concentrates within lysosomes, disrupting lipid domains and allowing molecules to leak across the membrane (Villamil Giraldo et al., 2014).

Cathepsin C (CTSC, also known as dipeptidyl peptidase 1) is widely supposed to be expressed mainly within lysosomes (Rao et al., 1997), where it cleaves a pair of N-terminal residues from its peptide substrates until it reaches a proline or basic residue. At neutral pH, cathepsin C can also polymerise dipeptides (McGuire et al., 1992; Thiele and Lipsky, 1990). These properties of cathepsin C have been exploited to allow selective disruption of lysosomes. For example, the esterified dipeptide, l-leucyl-l-leucine methyl ester (LLOMe, also known as Leu-Leu-OMe), disrupts lysosomes and, thereby, triggers apoptosis. The likely mechanism involves an initial lysosomotropic accumulation of LLOMe within lysosomes, causing pH_ly_ to increase; cathepsin C then catalyses polymerisation of the de-esterified dipeptide and, as the hydrophobic polymer accumulates, it perturbs the lysosomal membrane, rendering it permeable to small molecules (molecular mass<10 kDa) (Repnik et al., 2017; Thiele and Lipsky, 1990).

GPN (Fig. 1A), another synthetic substrate of cathepsin C, has been used extensively to perturb lysosomes, with ∼100 publications reporting its use (e.g. Berg et al., 1994; Churchill et al., 2002; Coen et al., 2012; Davis et al., 2012; Dionisio et al., 2011; Fois et al., 2015; Garrity et al., 2016; Haller et al., 1996; Kilpatrick et al., 2013; Li et al., 2012; Melchionda et al., 2016; Morgan and Galione, 2007; Penny et al., 2015, 2014; Ruas et al., 2015). It is assumed that GPN disrupts lysosomes because it is degraded within them by cathepsin C (hence, its selectivity for lysosomes); then the dipeptide accumulates, generating osmotic stress that ruptures lysosome membranes (Berg et al., 1994). There is evidence (from release of dextran-conjugated fluorophores) that modified dipeptides can rupture lysosomes (e.g. Repnik et al., 2017), but many more papers mistakenly assume that loss of Acridine Orange or LysoTracker signifies rupture of lysosomes (e.g. Uchimoto et al., 1999). However, Acridine Orange and LysoTracker, like other lysosomotropic agents, will redistribute across intact lysosomal membranes when pH_ly_ increases.

The widespread use of GPN to acutely assess the Ca^2+^ content of lysosomes and the lack of evidence that these effects are mediated by cathepsin C encouraged us to assess both the mechanism of action of GPN and its selectivity for lysosomes. We show that GPN evokes a rapid and sustained increase in pH_ly_, and transient increases in cytosolic pH (pH_cyt_) and cytosolic free Ca^2+^ concentration ([Ca^2+^]_c_). None of these responses requires cathepsin C activity; neither are they accompanied by detectable rupture of lysosome membranes. Rather than selectively stimulating Ca^2+^ release form lysosomes, GPN increases [Ca^2+^]_c_ by causing an increase in pH_cyt_ that then stimulates Ca^2+^ release from the ER by a mechanism that requires neither inositol 1,4,5-trisphosphate (IP_3_) receptors nor ryanodine receptors.

## RESULTS

### GPN evokes pH changes and Ca^2+^ signals without rupturing lysosomes

GPN is widely used to dissipate the lysosomal pH gradient and release Ca^2+^ from lysosomes (see Introduction) (Fig. 1A). In HEK cells, GPN caused a sustained increase in pH_ly_ and a transient increase in [Ca^2+^]_c_ (Fig. 1B-F, Fig. S1A,B). The Ca^2+^ signals evoked by GPN were slower than those evoked by carbachol, which stimulates IP_3_ formation and Ca^2+^ release through IP_3_ receptors (IP_3_Rs), but faster than those evoked by inhibiting the ER Ca^2+^ pumps (sarcoplasmic/endoplasmic reticulum Ca^2+^-ATPases, SERCAs) with cyclopiazonic acid (CPA) or thapsigargin (Fig. 1D,G, Fig. S1C,D). While these results are consistent with the reported actions of GPN, additional observations are not consistent with its presumed mode of action (Fig. 1A).

First, GPN caused a rapid and transient increase in cytosolic pH (pH_cyt_) (Fig. 1H). Rupture of acidic lysosomes would be expected to decrease pH_cyt_, as occurs, for example, when tumour necrosis factor alpha (TNFα) triggers loss of H^+^ and cathepsin D from lysosomes during the early stages of apoptosis (Nilsson et al., 2006). Similarly transient increases in pH_cyt_ were observed in other cell types and with different sources of GPN (Fig. S1E,F). Dissipation of the lysosomal pH gradient by inhibiting the V-ATPase with bafilomycin A_1_ had no effect on basal pH_cyt_ or the subsequent GPN-evoked increase in pH_cyt_ (Fig. 1I,J). These results show that H^+^ within lysosomes does not contribute to GPN-evoked changes in pH_cyt_. Since Ca^2+^ indicators are pH-sensitive (see Speake and Elliott, 1998), we confirmed that the affinity of fluo 8 for Ca^2+^ was unaffected by changing pH between 7 and 8 (Fig. S2), indicating that the GPN-evoked increases in fluo 8 fluorescence are due to an increase in [Ca^2+^]_c_ (Fig. 1D-F).

Second, the acute effects of GPN on pH_ly_ and pH_cyt_ were not accompanied by loss of endocytosed fluorescent molecules with molecular masses ranging from 447 (Lucifer Yellow), through ∼3 kDa (fluorescein-dextran) to ∼10 kDa (dextran conjugates of Alexa Fluor 488 and Oregon Green) (Fig. 1B,K,L, Fig. S1G). These results are not consistent with the effects of GPN on pH or [Ca^2+^]_c_ arising from rupture of lysosomes. More prolonged incubations with GPN (>10 min) caused some loss of lysosomal Alexa Fluor 488-dextran but, even after 25 min, most was retained by lysosomes (Fig. S3)

Third, the increase in [Ca^2+^]_c_ evoked by GPN was abolished by depleting the ER of Ca^2+^ by using CPA or thapsigargin to inhibit SERCAs (Fig. 1D,F). Thapsigargin had no effect on the GPN-evoked increases in pH_ly_ or pH_cyt_ (Fig. S1H,I). Although no SERCAs were detected in the proteome of lysosomal membranes (Bagshaw et al., 2005; Chapel et al., 2013), it has been suggested that a SERCA with reduced Ca^2+^ affinity, i.e. SERCA3, may contribute to Ca^2+^ uptake by acidic stores (López et al., 2005). We, therefore, considered whether inhibition of GPN-evoked cytosolic Ca^2+^ signals by thapsigargin and CPA is due to direct inhibition of lysosomal Ca^2+^ sequestration. However, IP_3_-evoked Ca^2+^ release from the ER also massively and rapidly attenuated the cytosolic Ca^2+^ signals evoked by GPN (Fig. S4). This confirms that loss of Ca^2+^ from the ER, rather than inhibition of SERCAs themselves, rapidly inhibits GPN-evoked increases in [Ca^2+^]_c_.

### GPN does not stimulate Ca^2+^-induced Ca^2+^ release from the ER

Others have suggested that GPN-evoked Ca^2+^ release from lysosomes is amplified by Ca^2+^-induced Ca^2+^ release (CICR) by IP_3_Rs or ryanodine receptors (RyRs) in the ER, and that this accounts for the inhibition of GPN responses by thapsigargin (Kilpatrick et al., 2013). Although HEK and HAP1 cells appear not to express functional RyRs (Fig. S5A,B) (Tong et al., 1999), we confirmed that ryanodine (to inhibit RyRs) had no effect on the increase in [Ca^2+^]_c_ evoked by GPN (Fig. S5C,D). Complete loss of IP_3_Rs in HEK (Alzayady et al., 2016) or HAP1 cells (Atakpa et al., 2018) by means of CRISPR/Cas9 had no effect on the Ca^2+^ signals evoked by GPN. In each case, the response to GPN was abolished by thapsigargin (Fig. 1F, Fig. S5E).

**Fig. 2.**
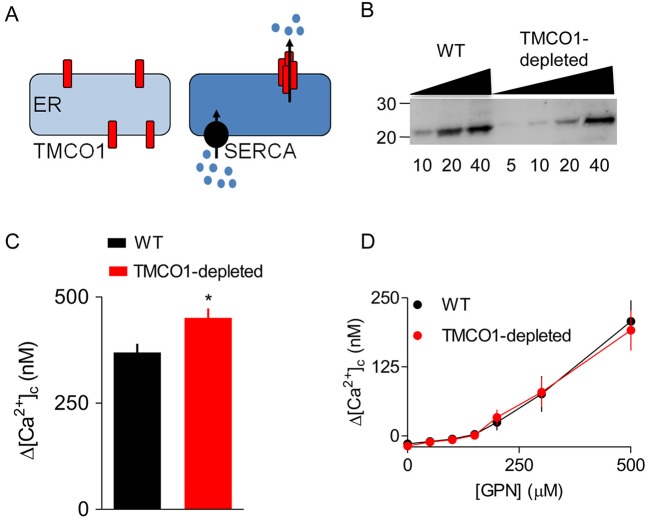
**CICR mediated by TMCO1 does not contribute to GPN-evoked increases in [Ca^2+^]_c_.** (A) TMCO1 has been proposed to oligomerise into a functional Ca^2+^-permeable channel as the ER loads with Ca^2+^ (Wang et al., 2016). Hence, TMCO1 might be able to mediate CICR because Ca^2+^ released by other intracellular channels fuels ER Ca^2+^ uptake by SERCAs, leading to Ca^2+^ overload and opening of TMCO1. (B) Typical western blot showing expression of TMCO1 in HEK cells in which CRISPR/Cas9 was used to achieve a partial knockdown of TMCO1 (67±14%, *n*=4, mean±s.d.). Molecular mass markers (kDa) and protein loadings (μg) are shown. (C,D) Peak Ca^2+^ signals evoked by a maximally effective concentration of carbachol (C) or the indicated concentrations of GPN (D) in Ca^2+^-free HBS (mean±s.e.m., *n*=3). *P<0.05, Student's *t*-test.

Transmembrane coiled-coil domain 1 (TMCO1) is an ER membrane protein that oligomerises to form a Ca^2+^ pore as the ER overloads with Ca^2+^ (Fig. 2A) (Wang et al., 2016). TMCO1 might, thereby, allow GPN-evoked Ca^2+^ release from lysosomes to be amplified. We failed, using CRISPR/Cas9, to achieve complete knockout of TMCO1 in HEK cells but, with TMCO1 expression reduced by 67±14% (Fig. 2B), we observed the expected increase in carbachol-evoked Ca^2+^ signals (Wang et al., 2016). However, there was no effect on the increase in [Ca^2+^]_c_ evoked by GPN (Fig. 2C,D). We, therefore, conclude that none of the known mechanisms (RyR, IP_3_R and TMCO1) that can mediate CICR from the ER contributed to the GPN-evoked increase in [Ca^2+^]_c_. The results, so far, demonstrate that GPN increases pH_ly_ and [Ca^2+^]_c_, but they challenge the conventional explanations of these phenomena.

### Effects of GPN on pH_ly_, pH_cyt_ and cytosolic Ca^2+^ do not require cathepsin C

**Fig. 3.**
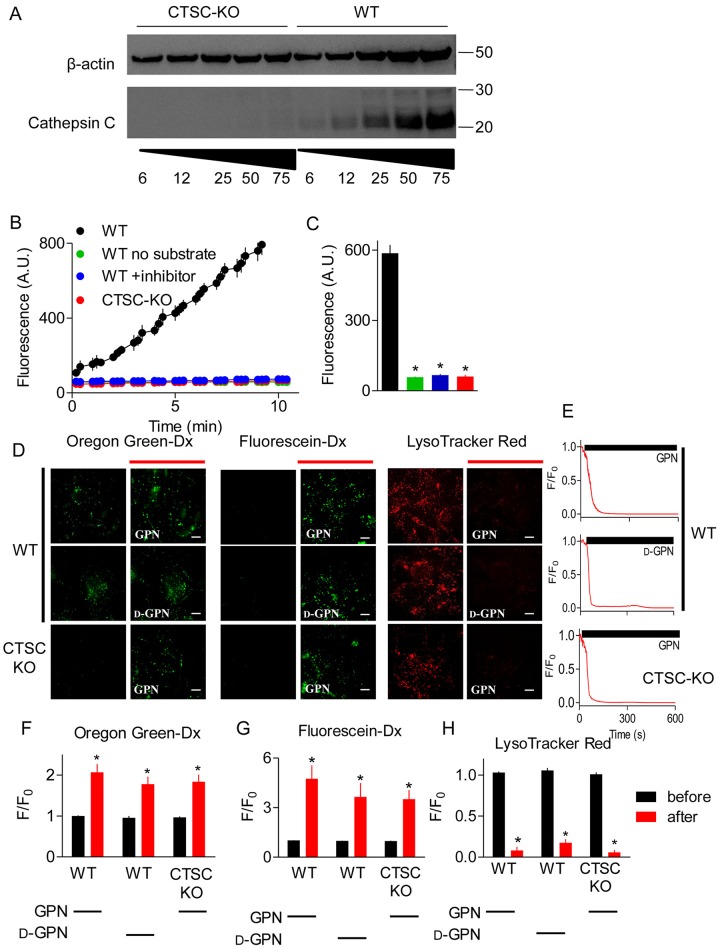
**Effects of GPN on pH_ly_ do not require cathepsin C.** (A) Western blot shows expression of cathepsin C and β-actin in wild-type (WT) HEK cells and after CRISPR/Cas9-mediated disruption of the cathepsin C genes (CTSC-KO). Protein loadings (µg) and positions of molecular mass markers (kDa) are shown. A representative for five similar blots is shown. (B) Cathepsin C activity, determined using a substrate (Gly-Arg-AMC) that fluoresces after proteolysis, was determined using whole-cell lysates from WT or CTSC-KO cells, alone or after treatment of cells with the cathepsin C inhibitor Gly-Phe-DMK (10 μM, 72 h, 37°C). Results show mean±s.d. for duplicate determinations. The green symbols are obscured by the overlying blue symbols. (C) Summary results (mean±s.e.m., *n*=3) from analyses similar to those in panel B show fluorescence recorded after 7 min. *P<0.05, one-way ANOVA, with Tukey's multiple comparison test (see panel B for colour key). (D) Lysosomes of WT or CTSC-KO HEK cells were loaded with pH indicators by endocytosis of dextran conjugates (Oregon Green and fluorescein) or by incubation with LysoTracker Red (100 nM, 20 min). Fluorescence was recorded before or 200 s after addition of GPN or d-GPN (300 µM each). Scale bars: 10 μm. (E) Time courses of the changes in LysoTracker Red fluorescence (F/F_0_) after addition of GPN or d-GPN (solid bars) in WT and CTSC-KO cells. (F-H) Summary results (mean±s.e.m., *n*=5) from analyses similar to those in panel E show the fluorescence changes (F/F_0_) before and 200 s after addition of the indicated GPN (300 µM). *P<0.05, Student's *t*-test relative to before GPN-treatment.

The effects of GPN are thought to require its proteolysis by cathepsin C (Fig. 1A), an enzyme that is widely assumed to be expressed mainly in lysosomes (Rao et al., 1997; Wolters and Chapman, 2000). This distribution of cathepsin C is invoked to suggest that GPN selectively targets lysosomes. Three approaches were adopted to assess the contribution of cathepsin C to the effects of GPN. We used CRISPR/Cas9 to generate HEK cells devoid of cathepsin C activity (HEK-CTSC-KO) (Fig. 3A-C). We used prolonged treatment with a selective peptide inhibitor of cathepsin C (Gly-Phe-DMK) (Méthot et al., 2007; Repnik et al., 2017), and demonstrated that it effectively inhibited enzyme activity in HEK cell lysates (Fig. 3B,C). We also used glycyl-d-phenylalanine (d-GPN), in which the l-phenylalanine of l-GPN (hitherto described as GPN) is replaced by d-phenylalanine, because d-GPN, unlike l-GPN, is not a substrate for cathepsin C (Jadot et al., 1990).

**Fig. 4.**
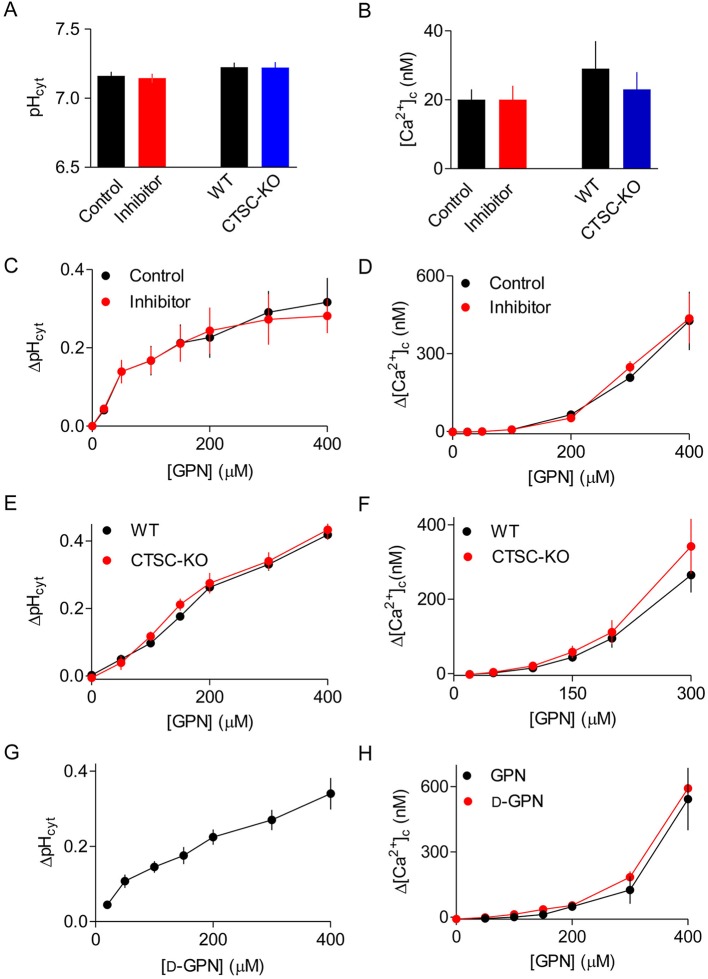
**Effects of GPN on pH_cyt_, pH_ly_ and [Ca^2+^]_c_ do not require cathepsin C.** (A,B) Effects of the cathepsin C inhibitor Gly-Phe-DMK (10 µM, 72 h) and comparison of WT with CTSC-KO cells on pH_cyt_ (A) and [Ca^2+^]_c_ (B) of unstimulated HEK cells. Matched controls (Control or WT) are shown for each comparison (inhibitor or CTSC-KO). (C,D) Effects of the cathepsin C inhibitor (Gly-Phe-DMK, 10 µM, 72 h) on the changes in pH_cyt_ (ΔpH_cyt_, measured 30 s after GPN addition) and the peak increase in [Ca^2+^]_c_ (Δ[Ca^2+^]_c_) evoked by GPN. (E,F) Results of analyses similar to those shown in C,D, comparing the effects of GPN in WT and CTSC-KO cells. (G,H) Results of analyses similar to those shown in C,D, comparing the effects of GPN and d-GPN. Results (A-H) show means±s.e.m., *n*=3, each with 3 replicates.

GPN caused the usual sustained increase in pH_ly_ in HEK cells without cathepsin C. Furthermore, in normal HEK cells, GPN and d-GPN caused indistinguishable increases in pH_ly_ (Fig. 3D-H). These results establish that the effects of GPN on pH_ly_ do not require cathepsin C activity. Neither loss of cathepsin C nor its pharmacological inhibition affected pH_cyt_ or the basal [Ca^2+^]_c_ in unstimulated cells (Fig. 4A,B). In addition, pharmacological inhibition of cathepsin C had no effect on the increase in pH_cyt_ or [Ca^2+^]_c_ evoked by addition of GPN (Fig. 4C,D). Furthermore, the concentration-dependent effects of GPN on pH_cyt_ and [Ca^2+^]_c_ were indistinguishable in HEK cells with and without endogenous cathepsin C (Fig. 4E,F). Finally, d-GPN, like GPN, caused the same concentration-dependent increases in pH_cyt_ and [Ca^2+^]_c_ as GPN (Fig. 4G,H).

LLOMe is a substrate of cathepsin C and, like GPN, it is a weak base but it is less membrane-permeable than GPN (Table S1). We confirmed that LLOMe caused lysis of lysosomes only in cells expressing cathepsin C (Fig. S6A), which is consistent with published work (Repnik et al., 2017). LLOMe also caused the expected increase in pH_ly_ but it minimally affected pH_cyt_ and had no effect on [Ca^2+^]_c_ (Fig. S6B-F). These results demonstrate that the effects of GPN on pH_ly_, pH_cyt_ and [Ca^2+^]_c_ do not require cathepsin C, whereas LLOMe causes cathepsin C-mediated lysis of lysosomes without increasing [Ca^2+^]_c_.

### Other weak bases evoke ER-dependent Ca^2+^ signals

**Fig. 5.**
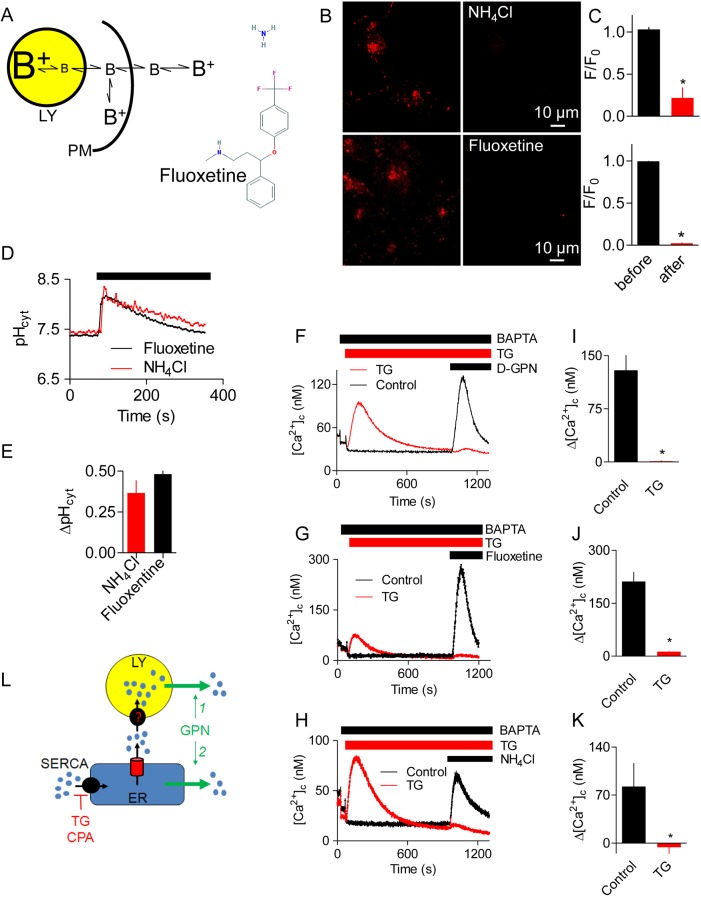
**Other weak bases evoke ER-dependent Ca^2+^ signals.** (A) Structures and mechanisms of action of some lysosomotropic drugs. (B) Confocal images of HEK cells loaded with LysoTracker Red (100 nM, 20 min) and then treated (200 s) with NH_4_Cl (20 mM) or fluoxetine (300 μM). (C) Quantification of fluorescence (F/F_0_) from images similar to those in panel B. Results show mean±s.e.m., *n*=7. F_0_ and F are fluorescence recorded before and 200 s after the addition of NH_4_Cl or fluoxetine. (D) Effects of NH_4_Cl (20 mM) or fluoxetine (300 μM) on pH_cyt_ recorded in populations of SNARF-5F-loaded HEK cells. Results show mean±s.d. of *n*=3 determinations. (E) Summary results (mean±s.e.m., *n*=3, each with 3 replicates) show peak increases in pH_cyt_ (ΔpH_cyt_). (F-H) Effects of thapsigargin (TG, 1 μM, 15 min) in Ca^2+^-free HBS on the Ca^2+^ signals evoked by d-GPN (200 μM) (F), fluoxetine (300 μM) (G) or NH_4_Cl (20 mM) (H). Results show mean±s.d. of *n*= 3 determinations. (I-K) Summary results from analyses similar to those in panels F-H show the effects of d-GPN (I), fluoxetine (J) or NH_4_Cl (K) on Δ[Ca^2+^]_c_ alone or after treatment with thapsigargin (TG, 1 μM, 15 min). Results show mean±s.e.m., *n*=3, each with 3 replicates. (L) A requirement for ER Ca^2+^ for GPN to evoke an increase in [Ca^2+^]_c_ might reflect a need for the ER to fuel lysosomal Ca^2+^ uptake (option 1) or a direct action of GPN on the ER (option 2).

GPN, but not d-GPN, is a substrate for cathepsin C; however, both are amphipathic weak bases (Table S1), which, in common with other lysosomotropic agents, accumulate within lysosomes (Fig. 5A) (Nadanaciva et al., 2011; Villamil Giraldo et al., 2014). We, therefore compared the actions of GPN with those of NH_4_Cl, another lysosomotropic agent that is often used to increase pH_ly_ (e.g. Johnson et al., 2016), and with fluoxetine (the antidepressant known as Prozac), an unrelated structure that has similar physical properties (p*K_a_* and hydrophobicity) to GPN (Fig. 5A and Table S1).

As expected NH_4_Cl and fluoxetine caused rapid and sustained increases in pH_ly_ (Fig. 5B,C), but they also caused transient increases in pH_cyt_ and [Ca^2+^]_c_, similar to those evoked by GPN (Fig. 5D-K). Furthermore, the Ca^2+^ signals evoked by NH_4_Cl and fluoxetine, like those evoked by GPN and d-GPN, were abolished by pretreatment with thapsigargin (Fig. 5F-K). The increases in [Ca^2+^]_c_ evoked by NH_4_Cl and fluoxetine were similar in cells with and without IP_3_Rs (Fig. S7A,B). By using carbachol or thapsigargin to estimate the residual Ca^2+^ content of the ER (through stimulation of IP_3_ formation or inhibition of SERCAs, respectively), we confirmed that both GPN and NH_4_Cl caused a concentration-dependent decrease in the ER Ca^2+^ content (Fig. S7C,D). These results establish that four different weak bases (GPN, d-GPN, NH_4_Cl and fluoxetine) have similar effects. Each causes a sustained increase in pH_ly,_ and transient increases in pH_cyt_ and [Ca^2+^]_c_. The latter invariably requires Ca^2+^ within the ER, but requires neither IP_3_Rs nor RyRs.

### GPN stimulates Ca^2+^ release from the ER by increasing pH_cyt_

Since no known CICR mechanism contributes to the Ca^2+^ signals evoked by GPN (Figs 1F and 2, Fig. S5), the requirement for ER Ca^2+^ cannot arise from it amplifying an initial GPN-evoked Ca^2+^ release from lysosomes. However, we (Atakpa et al., 2018; López Sanjurjo et al., 2013) and others (Garrity et al., 2016) have suggested that the ER also contributes to Ca^2+^ uptake by lysosomes (Fig. 5L). We, therefore, considered whether the primary action of GPN is to trigger Ca^2+^ release from lysosomes, which might be attenuated when lysosomes can no longer acquire Ca^2+^ from ER (Fig. 5L, option 1). Evidence that IP_3_-evoked Ca^2+^ release from the ER immediately attenuates responses to GPN (Fig. S4) argues against this proposal, and subsequent experiments confirm that it is untenable.

**Fig. 6.**
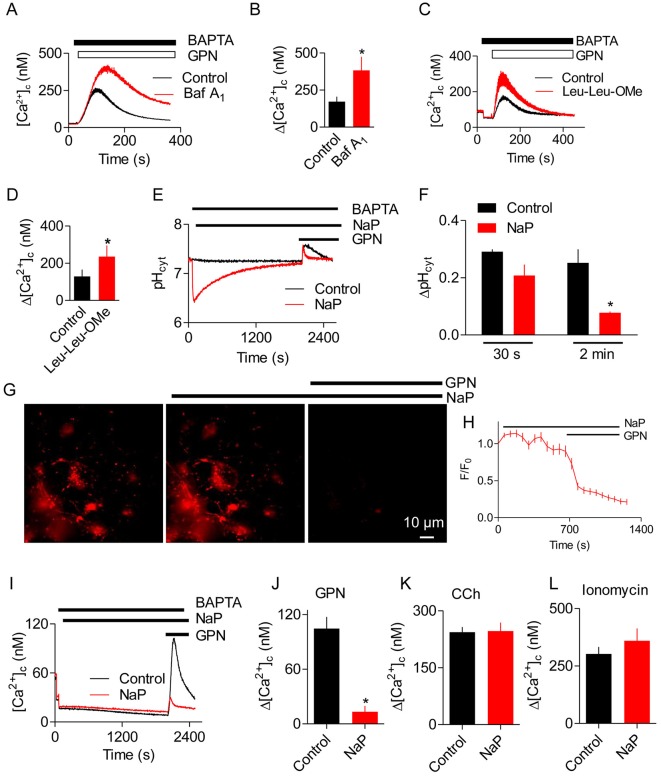
**Buffering the GPN-evoked increase in pH_cyt_ abolishes Ca^2+^ release from the ER.** (A) Effects of bafilomycin A_1_ (Baf A_1_, 1 μM, 1 h) on the Ca^2+^ signals evoked by GPN (200 μM) in HEK cells in Ca^2+^-free HBS. Mean±s.d. of 3 replicates. (B) Summary results (mean±s.e.m., *n*=4, each with 3 replicates) from analyses similar to those in panel A show peak increases in [ΔCa^2+^]_c_ evoked by GPN (Δ[Ca^2+^]_c_). *P<0.05, Student's *t*-test. (C) Effects of pre-treating HEK cells with LLOMe (Leu-Leu-OMe; 1 mM, 1 h) on the Ca^2+^ signals evoked by GPN (200 μM) in Ca^2+^-free HBS. Mean±s.d. of 3 replicates. (D) Summary results (mean±s.e.m., *n*=4, each with 3 replicates) from analyses similar to those in panel C show peak increases in [Ca^2+^]_c_ evoked by GPN (Δ[Ca^2+^]_c_). *P<0.05, Student's *t*-test. (E) HEK cells in Ca^2+^-free HBS were treated with sodium proprionate (NaP, 30 mM) before addition of GPN (200 μM). Results show pH_cyt_ (mean of 3 replicates). (F) Summary results (mean±s.e.m., *n*=3, each with 3 replicates) from analyses similar to those in panel E show pH_cyt_ measured 30 s or 2 min after GPN addition. *P<0.05, Student's *t*-test. (G) Typical images of HEK cells showing the effects of NaP (30 mM, 30 min) and then GPN (200 μM, 30 s) on LysoTracker Red staining. (H) Time-course (mean±s.d., *n*= 3 cells). (I) Analysis of the effects of NaP and GPN on [Ca^2+^]_c_ (mean of 3 replicates). (J-L) Summary results [mean±s.e.m., *n*=4 (J) or *n*=3 (K,L), each with 3 replicates] from analyses similar to those in panel E show peak Ca^2+^ signals (Δ[Ca^2+^]_c_) evoked by GPN (200 μM), carbachol (CCh, 1 mM) or ionomycin (5 μM) in Ca^2+^-free HBS. *P<0.05, Student's *t*-test.

After dissipation of the lysosomal pH gradient by sustained treatment with bafilomycin A_1_, the GPN-evoked increase in [Ca^2+^]_c_ was exaggerated (Fig. 6A,B), just as responses to Ca^2+^ release from the ER through IP_3_Rs or inhibition of SERCAs are exaggerated after treatment with bafilomycin A_1_ (Atakpa et al., 2018; López Sanjurjo et al., 2013). Similar results were obtained after lysis of lysosomes by pre-treatment with LLOMe, namely the increase in [Ca^2+^]_c_ evoked by GPN was exaggerated (Fig. 6C,D). Hence, under conditions where GPN cannot cause an increase in pH_ly_ and when lysosomes are unable to accumulate Ca^2+^ (Atakpa et al., 2018), GPN evokes larger increases in [Ca^2+^]_c_. We, therefore, considered whether the GPN-evoked increases in pH_cyt_ directly stimulate Ca^2+^ release from the ER (Fig. 5L*2*).

**Fig. 7.**
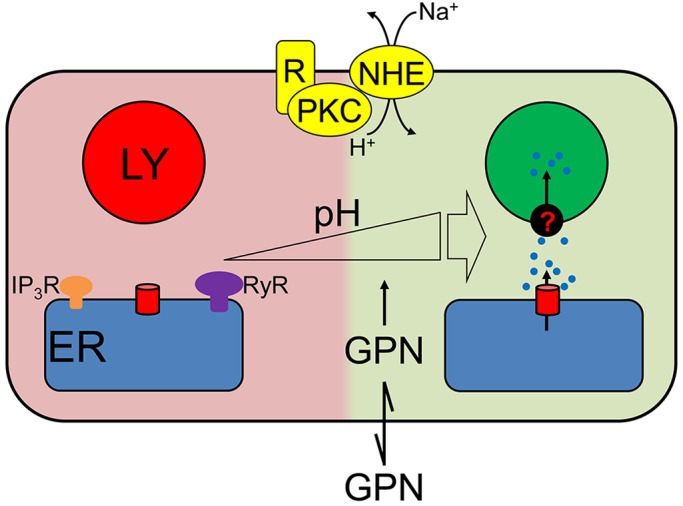
**GPN evokes Ca^2+^ release from the ER through an increase in pH_cyt_.** GPN, a weak membrane-permeant base, causes pH_cyt_ to transiently increase which directly stimulates Ca^2+^ release from the ER through a mechanism that requires neither IP_3_Rs nor RyRs. Some of the Ca^2+^ released by this pH-regulated mechanism, similarly to Ca^2+^ released by IP_3_Rs, is then accumulated by lysosomes (LY). Many physiological stimuli that increase pH_cyt_, including those that stimulate the Na^+^/H^+^ antiporter (NHE) through protein kinase C (PKC), evoke increases in [Ca^2+^]_c_ through the same pathway.

We loaded HEK cells with sodium proprionate (NaP), a weak acid, to buffer changes in pH_cyt_ evoked by GPN. NaP caused an initial drop in pH_cyt_ that slowly recovered, and subsequent addition of GPN caused an attenuated increase in pH_cyt_ (Fig. 6E). The immediate effect of GPN on pH_cyt_ was only modestly attenuated by NaP, but after 2 min the response was reduced by >60% (Fig. 6F). NaP did not affect pH_ly_ or prevent GPN from rapidly increasing pH_ly_ (Fig. 6G,H). The GPN-evoked increase in [Ca^2+^]_c_, which takes 106±2 s to reach its peak (Fig. 1G, Fig. S1C), was almost abolished by NaP (Fig. 6I,J). NaP had no effect on the increase in [Ca^2+^]_c_ evoked by carbachol or the Ca^2+^ content of the intracellular stores assessed by addition of ionomycin (Fig. 6K,L). These results demonstrate that an increase in pH_cyt_ is required for GPN to increase [Ca^2+^]_c_, whereas an increase in pH_ly_ is ineffective (Fig. 7).

## DISCUSSION

GPN is used extensively to perturb lysosomes and, as interest in the contributions of lysosomes to Ca^2+^ signalling has grown, has been widely used to release Ca^2+^ from lysosomes (see Introduction). It has been universally assumed that GPN achieves selectivity for lysosomes because its cleavage by the lysosomal enzyme cathepsin C causes osmotic stress that ruptures lysosomal membranes (Fig. 1A). Our results challenge these assumptions and demonstrate that GPN does not selectively target lysosomes.

We have shown that GPN does not acutely rupture lysosomes (Fig. 1B,C,K,L, Fig. S3) and that it causes a transient increase in pH_cyt_ (Fig. 1I,J, Fig. S1E,F). GPN also increases pH_ly_ and [Ca^2+^]_c_. None of these effects of GPN require cathepsin C (Figs 3 and 4). The GPN-evoked increase in [Ca^2+^]_c_ is mediated by Ca^2+^ release from the ER, without any evident involvement of lysosomes: Ca^2+^ signals are abolished by depleting the ER of Ca^2+^ (Figs 1D,F and 5F-K, Figs S4 and S5E), amplified when lysosomes are no longer acidified (Fig. 6A,B), and show no requirement for CICR from the ER (Figs 1F and 2, Fig. S5).

Several lines of evidence show that the GPN-evoked increase in pH_cyt_ stimulates Ca^2+^ release from the ER (Fig. 7). Other membrane-permeant weak bases mimic GPN (Fig. 5, Fig. S7), depletion of the ER of Ca^2+^ abolishes the GPN-evoked increase in [Ca^2+^]_c_ (Figs 1D-F and 5F-K, Figs S4 and S5E) without affecting the pH_cyt_ increase (Fig. S1I), and an increase in pH_ly_ is not required for GPN to release Ca^2+^ (Fig. 6A,B). Furthermore, rupture of lysosomes with LLOMe exaggerates, rather than prevents, cytosolic Ca^2+^ signals evoked by GPN (Fig. 6C,D, Fig. S6). Finally, GPN-evoked Ca^2+^ release is almost abolished when the pH_cyt_ changes are buffered under conditions where GPN still causes an increase in pH_ly_ (Fig. 6). We conclude that GPN, and other membrane-permeant weak bases, cause an increase in pH_cyt_, which then stimulates Ca^2+^ release from the ER (Fig. 7).

Cells tightly control their pH_cyt_, passively with buffers and actively by means of ion transporters (Casey et al., 2010), but many stimuli cause acute increases in pH_cyt_ at least as large as those evoked by GPN (ΔpH=0.37±0.06, Fig. 1H). The stimuli include those that activate the plasma membrane Na^+^-H^+^ exchanger (NHE) through protein kinase C (Wakabayashi et al., 2006) and signals that prepare sperm for fertilisation (Babcock et al., 1983). Furthermore, in tumours, pH_cyt_ is often increased (Schreiber, 2005). In many cell types, an increase in pH_cyt_ increases [Ca^2+^]_c_. In some cells, the response requires Ca^2+^ entry (Grinstein and Goetz, 1985; Wakabayashi et al., 2006) but in many others it is due to Ca^2+^ release from intracellular stores, most likely the ER (Batlle et al., 1993; Shorte et al., 1991; Siskind et al., 1989; Speake and Elliott, 1998; Willoughby et al., 2001; Yodozawa et al., 1997). The mechanisms are unknown and, since the pH of the ER lumen (pH_ER_) is assumed to equilibrate rapidly with pH_cyt_ (Casey et al., 2010), the effect might be exercised from the cytosol or ER lumen. Because increased pH_ER_ would be expected to increase Ca^2+^ buffering and so decrease ER luminal free [Ca^2+^], the Ca^2+^ release evoked by increased pH_cyt_ is probably due to the opening of a Ca^2+^-permeable ER channel. At increased pH_cyt_, phospholipase C may be stimulated (Yodozawa et al., 1997) and IP_3_ also binds more tightly to IP_3_Rs (Joseph et al., 1989), but GPN-evoked Ca^2+^ release does not require IP_3_Rs (Fig. 1F, Fig. S5E). SERCAs, which transport Ca^2+^ in exchange for H^+^, are inhibited by increased pH (Li et al., 2012; Lytton et al., 1992). However, inhibition of SERCAs cannot explain our results because the Ca^2+^ release evoked by GPN was both faster and more substantial than that evoked by inhibition of SERCAs by CPA or thapsigargin (Fig. 1D,G, Fig. S1D). Hence, modest increases in pH_cyt_, similar to those evoked by many physiological stimuli, trigger a substantial Ca^2+^ release from the ER that requires neither RyRs nor IP_3_Rs.

We conclude that GPN does not, as hitherto supposed, evoke Ca^2+^ release from lysosomes through its cathepsin C-mediated proteolysis. Instead, GPN, in common with other membrane-permeant weak bases and many physiological stimuli, transiently increases pH_cyt_, and that directly stimulates Ca^2+^ release from the ER by a mechanism that is independent of known ER Ca^2+^ release channels.

## MATERIALS AND METHODS

### Materials

SNARF-5F, dextran-conjugates of Oregon Green 488 (*M*_r_ 10,000), Alexa Fluor-488 (*M*_r_ 10,000 and 3000) or fluorescein (*M*_r_ 3000), LysoTracker Red DND-99, Lucifer Yellow, Platinum Pfx DNA polymerase, Tris-acetate SDS running buffer, 4–12% Bis-Tris polyacrylamide gels, iBLOT transfer kit, custom primers, EDTA-free Pierce protease inhibitor mini-tablets, Rapid DNA ligation kit, and Spectra multicolour broad range protein ladder were from ThermoFisher (Waltham, MA). Glycyl-l-phenylalanine 2-naphthylamide (GPN) was from Bachem (St. Helens, UK) and Santa Cruz Biotechnology (Dallas, TX); most experiments used GPN from Bachem (see Fig. S1E,F). Glycyl-d-phenylalanine 2-naphthylamide (d-GPN) was custom-synthesised by LifeTein (Somerset, NJ). Gly-Arg-7-amino-4-methylcoumarin (Gly-Arg-AMC) was from Bachem. Gly-Phe-diazomethylketone (Gly-Phe-DMK) was from MP Bio (Derby, UK). Fibronectin was from Merck Millipore (Watford, UK). ECL prime western blotting detection reagent was from GE Life Sciences (Little Chalfont, UK). Thapsigargin and cyclopiazonic acid (CPA) were from Bio-Techne (Minneapolis, MN). Fluo 8-AM was from Stratech Scientific (Suffolk, UK). 1,2-bis(O-aminophenoxy)ethane-N,N,N′,N′-tetraacetic acid (BAPTA) was from Phion (Dorset, UK). Ionomycin was from Apollo Scientific (Stockport, UK). Ryanodine, Pluronic F-127, sodium propionate (NaP), fluoxetine hydrochloride, Leu-Leu methyl ester hydrobromide (Leu-Leu-OMe) and carbamylcholine chloride (carbachol, CCh) were from Sigma (Dorset, UK).

### Cell culture

HEK and HeLa cell lines were cultured in Dulbecco's modified Eagle’s medium (DMEM)/F-12 with GlutaMAX (ThermoFisher) supplemented with foetal bovine serum (FBS, 10%, Sigma) at 37°C in humidified air with 5% CO_2_. HEK cells lacking all IP_3_R subtypes (HEK-IP_3_R-KO) were from Kerafast (Boston, MA) (Alzayady et al., 2016). HAP1 cells, genetically engineered using CRISPR/Cas9 to disrupt genes for all three IP_3_R subtypes, were developed with Horizon Discovery (Cambridge, UK) (Atakpa et al., 2018). HAP1 cells were cultured in Iscove's modified Dulbecco's medium (IMDM) GlutaMAX (ThermoFisher) with 10% FBS. The cells were maintained at 37°C in humidified air with 5% CO_2_. Cells were passaged every 3–4 days using GibcoTrypLE Express (ThermoFisher). All cell lines were confirmed to be free of mycoplasma.

For imaging, cells were grown on glass-bottomed dishes (35-mm with a 7-mm No. 0 glass insert, MatTek Corporation, Ashland, MA) coated with human fibronectin (10 µg/ml). BacMam baculovirus encoding human M_3_ muscarinic acetylcholine receptors (M_3_Rs) was produced as described (Taylor et al., 2017). HEK cells were infected at a multiplicity of infection (MOI) of ∼50 and used after 48 h (Fig. S4).

### Generation of HEK cells without cathepsin C

CRISPR/Cas9 was used to disrupt the genes encoding cathepsin C (CTSC). Guide sequences were selected using E-crispr.org (Heigwer et al., 2014) and zitfit.partners.org (Sander et al., 2010). Oligonucleotides encoding four different guide RNAs (sgRNA) were used to target the first, second and third exons of CTSC (sgRNAs for exon 1: 5′-GCTGGGCACCTGGGTCTTCC-3′ and 5′-GCCCTCCTGCTGCTTCTCTC-3′; sgRNA for exon 2: GATACAGCATATGATGACCT-3′; sgRNAs for exon 3: 5′-GTTGACATACACATTCTCAG-3′). Each guide and its complementary sequence had a sticky end (5′-CACC-3′ or 5′-AAAC-3′) to allow ligation into the Px458 vector, which also encodes Cas9 nuclease and GFP (Addgene #48138) (Ran et al., 2013). The coding sequences of the four final plasmids were verified. HEK cells were transfected with pX458-sgRNAs using Trans LT1 (4 µg DNA per 6-well plate). After 48 h, EGFP-expressing cells were sorted as single cells into 96-well plates by fluorescence-activated cell sorting (FACS). After 8 weeks, clones were screened by western blotting. The HEK-CTSC-KO cells express no detectable cathepsin C activity (Fig. 3A-C).

### Generation of HEK cells deficient in TMCO1

The methods used to generate cells deficient in TMCO1 were the same as those used to generate HEK-CTSC-KO cells. The only effective sgRNA (5′-GAAGCGGAAGTGCGATCTTC-3′) targeted exon 1. Neither different sgRNAs, targeting exon 1 (5′-GTGCACGGCTCTGCTCGCAG-3′) or exon 3 (5′-GAAACAATAACAGAGTCAGC-3′ and 5′-GTTTACAGTGGAAAAGAAGA-3′), nor repeated transfections with multiple sgRNAs succeeded in yielding cells with both TMCO1 alleles disrupted (Fig. 2).

### Measurements of pH_cyt_ in cell populations

Confluent cultures of cells grown in 96-well plates (Greiner Bio-One, Storehouse, UK) were loaded with the ratiometric pH indicator SNARF-5F (Liu et al., 2001) by incubation (30 min, 20°C) with SNARF-5F AM (2 µM, 30 min) in HEPES-buffered saline (HBS) containing Pluronic F-127 (0.02%). HBS had the following composition: 135 mM NaCl, 5.9 mM KCl, 1.2 mM MgCl_2_, 1.5 mM CaCl_2_, 11.5 mM glucose, 11.6 mM HEPES pH 7.3. Cells were washed in HBS, and fluorescence was recorded from cells in HBS at 20°C at intervals of 3.8 s (excitation, 543 nm; emission, 580 and 640 nm) using a FlexStation 3 fluorescence plate-reader with SoftMaxPro software (MDS Analytical Technologies, Wokingham, UK). Fluorescence was calibrated to pH_cyt_ from

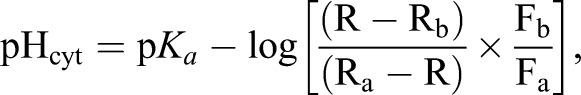
where, p*K_a_* is the negative log of the acid-base dissociation constant (*K_a_*); R is the fluorescence emission ratio (F_580_:F_640_); R_a_ (F_a_) and R_b_ (F_b_) are the fluorescence ratios (or fluorescence intensities at 640 nm) for the fully protonated and de-protonated forms of the indicator.

To determine the p*K_a_* of SNARF-5F, cells loaded with SNARF-5F were treated for 30 min in Ca^2+^-free cytosol-like medium (CLM pH 7.4) with the H^+^/K^+^ antiporter nigericin (50 µM). CLM was then replaced by CLM supplemented with nigericin (50 µM) and buffered at different pH values (pH 5.0–8.5), and SNARF-5F fluorescence was measured. Ca^2+^-free CLM had the following composition: KCl 140 mM, NaCl 4 mM, MgCl_2_ 1.4 mM, HEPES 10 mM, EGTA 1 mM. The resulting calibration plot of pH versus 
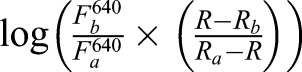
 had a slope of ∼1 and the intercept was the p*K*_a_ (7.09) used in all further calibrations.

### Measurements of [Ca^2+^]_c_ in cell populations

For measurements of cytosolic free [Ca^2+^] ([Ca^2+^]_c_), confluent cell monolayers grown in 96-well plates were loaded with fluo 8 by incubation for 1 h at 20°C in HBS (100 μl) containing fluo 8-AM and 0.02% Pluronic F-127. Cells were then washed and incubated in HBS for 1 h at 20°C before use. CaCl_2_ was omitted from nominally Ca^2+^-free HBS, and in some experiments (see figure legends) BAPTA (final concentration 2.5 mM) was added to the HBS immediately before stimulation to reduce the free [Ca^2+^] of the HBS to <20 nM. Fluorescence was recorded using a FlexStation 3 fluorescence plate-reader. Fluorescence was recorded at 1.44-s intervals, with excitation at 485 nm and emission at 525 nm. Data were collected and analyzed using SoftMax Pro software. Maximal (F_max_) and minimal (F_min_) fluorescence values were determined from parallel wells after addition of Triton X-100 (0.1%) with either 10 mM CaCl_2_ (F_max_) or 10 mM BAPTA (F_min_). Fluorescence values (F) were calibrated to [Ca^2+^]_c_ using a *K*_d_=389 nM (fluo 8) from

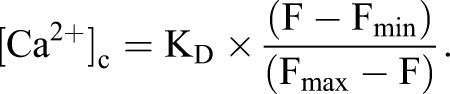


### Loading lysosomes with fluorescent indicators

To load lysosomes by endocytosis with fluorescent dyes, cells grown on poly-l-lysine-coated, glass-bottomed 35-mm dishes were incubated (16 h, 37°C) in culture medium supplemented with either a dextran-conjugated indicator (0.1 mg/ml) or Lucifer Yellow (0.2 mg/ml). After a further 4–6 h in the same medium without indicator, cells were used for experiments. For HEK cells expressing LAMP1-mCherry (Fig. S3) (López Sanjurjo et al., 2013), confluent cells were transfected using TransIT-LT1 reagent (2.5 µg DNA in 7.5 µl reagent per 35-mm dish; GeneFlow, Lichfield, UK) at the same time as they were loaded with dextran-conjugated Alexa Fluor 488 (*M*_r_ ∼10,000). For labelling with LysoTracker Red, cells were incubated in HBS with LysoTracker Red DND-99 (100 nM, 20°C). After 20 min, cells were washed three times with HBS and used immediately.

### Fluorescence microscopy

Fluorescence microscopy used inverted Olympus IX83 microscopes with 100× objectives (numerical aperture, NA 1.45 or 1.49), a multi-line laser bank (488 and 561 nm) and an iLas^2^ targeted laser illumination system (Cairn, Faversham, UK). Excitation light was transmitted through a quad dichroic beam-splitter (TRF89902-QUAD), and emitted light was passed through appropriate filters (Cairn Optospin). Wide-field images were collected with either an iXon Ultra 897 EMCCD camera (Andor, Belfast, Northern Ireland) or a 95B Scientific CMOS camera (Photometrics, Tuczon, AZ, USA) and MetaMorph microscopy automation and image analysis software (Molecular Devices). Bright-field images were acquired using a Cairn MonoLED illuminator.

Fluorescence from Oregon Green, fluorescein and Alexa Fluor 488 was excited at 488 nm and captured using a 525/50-nm filter (peak/bandwidth). LysoTracker Red and mCherry were excited at 561 nm and captured at 630/75 nm. All fluorescence images were corrected for background by subtraction of fluorescence from a region outside the cell.

### Analysis of fluorescence images

Time-lapse recordings of cells loaded with dextran-conjugated indicators were analyzed by taking randomly selected regions of interest (ROI) large enough for each to include several lysosomes (ROI, 3.2 µm×3.2 µm). Fluorescence changes were then expressed as F/F_0_, where F_0_ and F denote the average fluorescence within the ROI at the start of the experiment (F_0_) and at each subsequent time point (F).

To analyse the number of lysosomes in cells before and after GPN treatment (Fig. 1L), the Fiji TrackMate plugin (Tinevez et al., 2017) was used to identify lysosomes as spots in background-corrected wide-field images.

For analysis of Alexa Fluor 488-dextran distribution in cells expressing LAMP1-mCherry, the FFT bandpass filter and thresholding functions in Fiji (Schindelin et al., 2012) were used to generate a binary image to identify ROIs expressing LAMP1-mCherry (10–1000 pixels with a circularity of 0.1–1). Within the identified ROIs, the background-corrected fluorescence images for LAMP1-mCherry and Alexa Fluor 488 were used to compute a fluorescence ratio for each ROI (F_488_:F_561_). The F_488_:F_561_ values for all ROIs within each cell (typically 70–270, but ranging from 33–486) were averaged for each image, and then expressed relative to the value determined before treatment (Fig. S3). This averaging within a cell was required because lysosomes move during the 25-min recording interval, making it impossible to track individual ROIs in recordings that were collected at 5-min intervals (to minimise photobleaching).

### Western blots

Cells in 6-well plates were washed, lysed in RIPA buffer (30 μl, 4°C, 1 h) by using a syringe needle and sonication, and the supernatant (14,000 ***g***, 15 min, 4°C) was used for analysis.

RIPA buffer comprised: 150 mM NaCl, 1% Triton X-100, 0.5% sodium deoxycholate, 0.1% SDS, 50 mM Tris pH 8. Protein samples were separated on 4–12% Bis-Tris PAGE gels, transferred to a polyvinyl difluoride (PVDF) membrane using an iBLOT system, and blocked by incubation in TBST with gentle shaking (1 h, 20°C). Tris-buffered saline (TBS) comprised: 150 mM NaCl, 50 mM Tris pH 7.5. For TBST, TBS was supplemented with 0.1% Tween-20 and 5% BSA. The membrane was washed with TBST, incubated with primary antibody (16 h, 40°C), washed with TBST (3×5 min, 20°C), incubated with HRP-conjugated secondary antibody in TBST with 1% BSA (1 h, 20°C), and washed with TBST (3×5 min, 20°C). Enhanced chemiluminescence (ECL) primer western blotting detection reagent (Amersham, UK) and a Syngene PXi chemiluminescence detection system were used to detect HRP. The antibodies used were mouse anti-cathepsin C (Santa Cruz Inc., Cat# s74590, 1:500), rabbit anti-TMCO1 (Sigma, Cat# AV49429, 1:1000), mouse anti β-actin (Cell Signaling, Cat# 8H10D10, 1:1000), donkey anti-mouse IgG-HRP (Santa Cruz Inc., Cat# sc-2314, 1:2000) and donkey anti-rabbit IgG-HRP (Santa Cruz Inc., Cat# sc-2313, 1:5000).

### Measurement of cathepsin C activity

Cathepsin C activity was measured by using a substrate, Gly-Arg-AMC, that becomes fluorescent after its proteolytic cleavage (Eick et al., 2014; Hamilton et al., 2008). HEK cell lysates were diluted in assay buffer (25 mM MES, 50 mM NaCl, 5 mM DTT, pH 6) to provide a protein concentration of 200 µg/ml, and distributed into 96-well plates (50 µl/well). Cleavage of Gly-Arg-AMC (50 µM) was monitored at 0.2-min intervals (excitation, 380 nm; emission 460 nm) using a FlexStation 3 plate-reader and SoftMax Pro software. A substrate blank was included as a control.

### Statistical analysis

Results are presented as means±s.e.m. or ±s.d., with *n* describing the number of independent analyses. Student's *t*-test, one-way or two-way ANOVA with Tukey's or Bonferroni test were used to determine statistical significance. *P*<0.05 was considered statistically significant.

## Supplementary Material

Supplementary information
