# Supplementary material for: GPN does not release lysosomal Ca2+ but evokes Ca2+ release from the ER by increasing the cytosolic pH independently of cathepsin C
Source: J Cell Sci. 2019 Feb 1;132(3):jcs223883. doi: 10.1242/jcs.223883 (PMC6382017; doi:10.1242/jcs.223883)
Supplement: Supplementary information [file joces-132-223883-s1.pdf]

SUPPLEMENTARY INFORMATION

GPN does not release lysosomal Ca^{2+}, but evokes Ca^{2+} release from the ER by increasing cytosolic pH independent of cathepsin C

Peace Atakpa, Laura M. van Marrewijk, Michael Apta-Smith, Sumita Chakraborty and Colin W. Taylor

Abbreviations

$[Ca^{2+}]_c$, cytosolic free Ca^{2+} concentration; CICR, Ca^{2+}-induced Ca^{2+} release; CPA, cyclopiazonic acid; CTSC, cathepsin C; ER, endoplasmic reticuum; GPN, glycyl-L-phenylalanine 2-napthylamide (D-GPN has D-phenylalanine); IP_3R, inositol 1,4,5-trisphosphate receptor; NaP, sodium proprionate; pH_{cyt} (pH_{ly}, pH_{ER}) cytosolic (lysosomal, ER) pH; RyR, ryanodine receptor; SERCA, SR/ER Ca^{2+}-ATPase.

Journal of Cell Science • Supplementary information

Table S1. Properties of GPN, fluoxetine and established lysosomotropic agents.

	[a]Estimated pK_a	[a]Estimated ACD/logP	[d]Estimated *C*logP
NH_3	8.86	-0.98	ND
LysoTracker Red	[b]7.5	ND	[b]2.10
[c]GPN	7.84	2.40	3.01
Fluoxetine	9.8	4.17	4.57
Leu-Leu-OMe	8.43	2.01	1.54

Basic pK_a refers to the pK_a of the most basic moiety within the molecule. logP is the log of the partition coefficient of the neutral species between n-octanol and water. ACD/logP and *C*log*P* are derived from different algorithms used to estimate logP values (reviewed in Mannhold et al., 2009). [a]From Chemicalize (2018) calculation module: https://chemicalize.com/ developed by ChemAxon (http://www.chemaxon.com); except for [b](Duvvuri et al., 2004). [c]For GPN, we disregard the most extremely basic moieties since their pK_a values (13.6 and 15.3) lie too far beyond the physiological pH range; pK_a values determined using the ACD/PhysChem Suite, version 12.0, Advanced Chemistry Development Inc, Toronto, Canada (http://ilab.cds.rsc.org/?cdsrdr=1). [d]From http://ilab.cds.rsc.org/?cdsrdr=1, except for [b](Duvvuri et al., 2004). Most lysosomotropic compounds have basic pK_a values >6.5 and *C*log*P* values >2 (Nadanaciva et al., 2011). ND, not determined.

SUPPLEMENTARY REFERENCES

Duvvuri, M., Gong, Y., Chatterji, D. and Krise, J. P. (2004). Weak base permeability characteristics influence the intracellular sequestration site in the multidrug-resistant human leukemic cell line HL-60. *J. Biol. Chem.* **279**, 32367-32372.

Konieczny, V., Tovey, S. C., Mataragka, S., Prole, D. L. and Taylor, C. W. (2017). Cyclic AMP recruits a discrete Intracellular Ca^{2+} store by unmasking hypersensitive IP_3 receptors. *Cell Rep.* **18**, 711-722.

Mannhold, R., Poda, G. I., Ostermann, C. and Tetko, I. V. (2009). Calculation of molecular lipophilicity: State-of-the-art and comparison of log P methods on more than 96,000 compounds. *J. Pharm. Sci.* **98**, 861-893.

Nadanaciva, S., Lu, S., Gebhard, D. F., Jessen, B. A., Pennie, W. D. and Will, Y. (2011). A high content screening assay for identifying lysosomotropic compounds. *Toxicol. In Vitro* **25**, 715-723.

Pethig, R., Kuhn, M., Payne, R., Adler, E., Chen, T. H. and Jaffe, L. F. (1989). On the dissociation constants of BAPTA-type calcium buffers. *Cell Calcium* **10**, 491-498.

Tsien, R. Y. (1980). New calcium indicators and buffers with high selectivity against magnesium and protons: design, synthesis, and properties of prototype structures. *Biochemistry* **19**, 2396-2404.

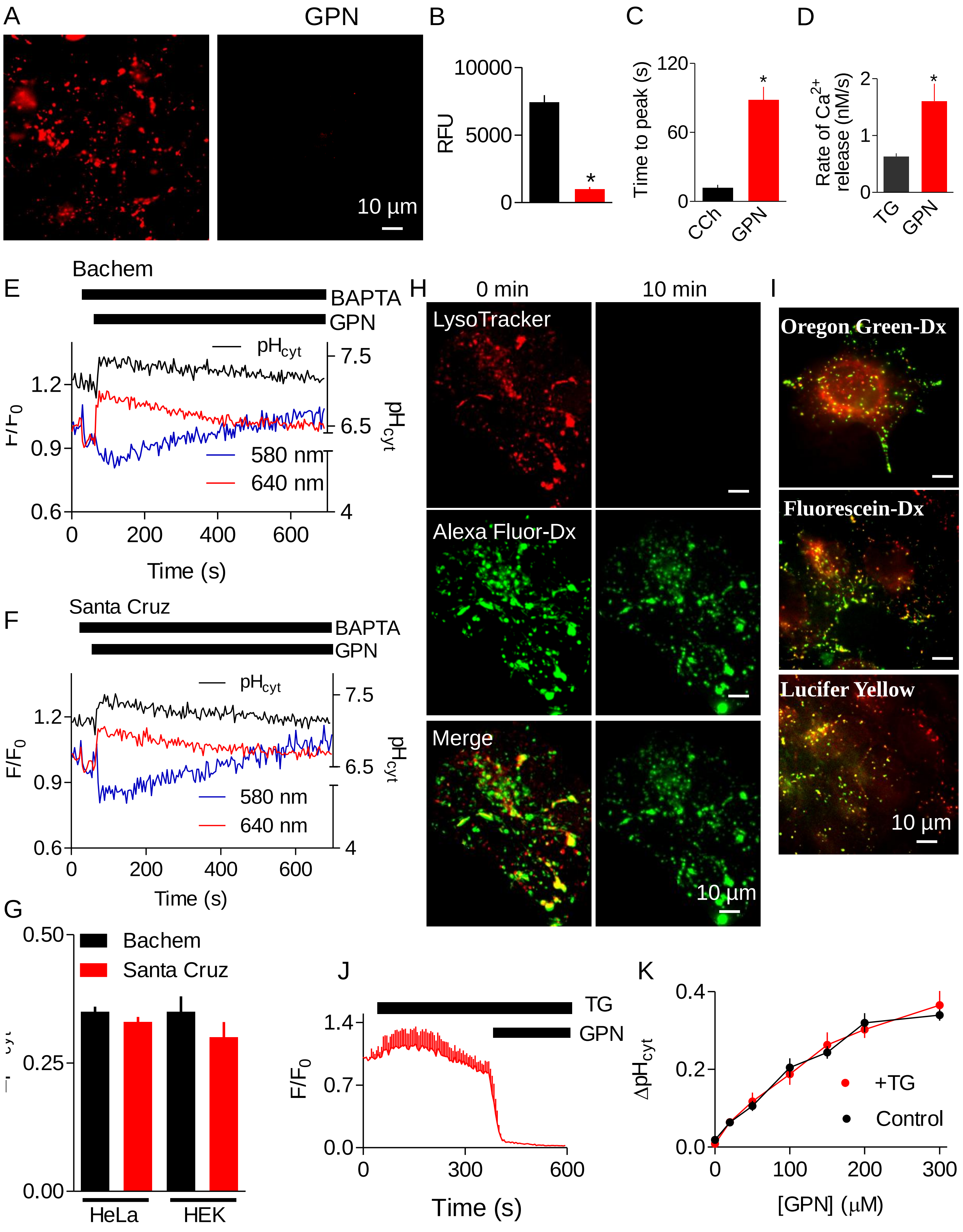

Journal of Cell Science • Supplementary information

Fig. S1. Similar responses to GPN from different sources and in different cells. Figure on preceding page. (**A**) Wide-field images show LysoTracker Red staining of HEK cells with and without GPN (200 μM, 30 min). The results show that even after prolonged stimulation with GPN, pH_{ly} does not recover. (**B**) Summary results (mean ± SEM, $n = 4$ coverslips) show the intensity of LysoTracker Red staining. (**C**) From experiments similar to those in **Fig. 1G**, times to peak increase in $[Ca^{2+}]_c$ are shown (mean ± SEM, $n = 4$, with 3 replicates). $^*P < 0.05$, Student's *t*-test. (**D**) Similar analysis shows rates of rise of $[Ca^{2+}]_c$ after addition of GPN (200 μM) or thapsigargin (1 μM) in Ca^{2+}-free HBS, each measured over the same range of $[Ca^{2+}]_c$. Results show means ± SEM, $n = 5$, with 3 replicates. $^*P <$ 0.05, Student's *t*-test. (**E,F**) Typical traces show the reciprocal changes in SNARF-5F fluorescence at the two emission wavelengths used (580 nm and 640 nm) and pH_{cyt} after addition of GPN (200 μM) from Bachem (E) or Santa Cruz (F). (**G**) Summary results (mean ± SEM, $n = 3$, with 3 replicates) show the effects of GPN (200 μM, from the indicated suppliers) on ΔpH_{cyt} of HEK and HeLa cells. (**H**) Simultaneous recording of LysoTracker Red and Alexa Fluor 488-Dx (M_r ~10,000) from HEK cells treated with GPN (200 μM, 10 min) showing that an increase in pH_{ly} is not accompanied by loss of large molecules from lysosomes. (**I**) Overlay images of HEK cells loaded with LysoTracker Red and either Oregon Green-Dx, Fluorescein-Dx or Lucifer Yellow. (**J**) Typical time course showing effect of GPN (200 μM) on LysoTracker Red fluorescence in HEK cells after treatment with thapsigargin (TG, 1 μM, 5 min). See **Fig. 1C** for a similar analysis without TG. (**K**) Similar analyses of the effects of the indicated concentrations of GPN in Ca^{2+}-free HBS alone or after treatment with thapsigargin (TG, 1 μM, 15 min) on ΔpH_{cyt} (mean ± SEM, $n = 3$, each with duplicate determinations).

Journal of Cell Science • Supplementary information

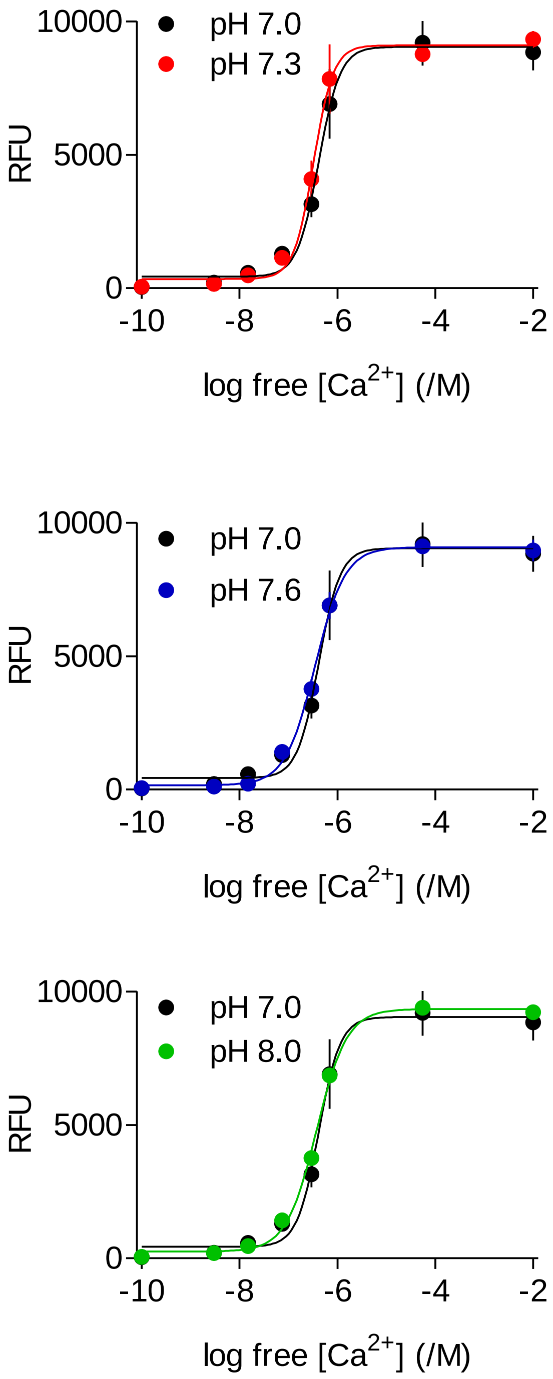

Fig. S2. The affinity of fluo 8 for Ca^{2+} is unaffected by changing pH between 7 and 8. Since the K_d of BAPTA for Ca^{2+} (160 nM in the absence of Mg^{2+}) (Pethig et al., 1989) is unaffected by pH changes between 7 and 8 (Tsien, 1980), the free [Ca^{2+}] of cytosol-like medium (CLM) containing 10 mM BAPTA, but without Mg^{2+}, was computed at the indicated pH using the same K_d for Ca^{2+} for each pH. Results (mean ± SEM, *n* = 3, each with 3 determinations) show the fluorescence recorded from fluo 8 (2 µM). RFU, relative fluorescence units. The results indicate that the K_d of fluo 8 for Ca^{2+} is the same at pH 7 (434 ± 51 nM), pH 7.3 (374 ± 43 nM), pH 7.6 (355 ± 39 nM) and pH 8 (401 ± 29 nM).

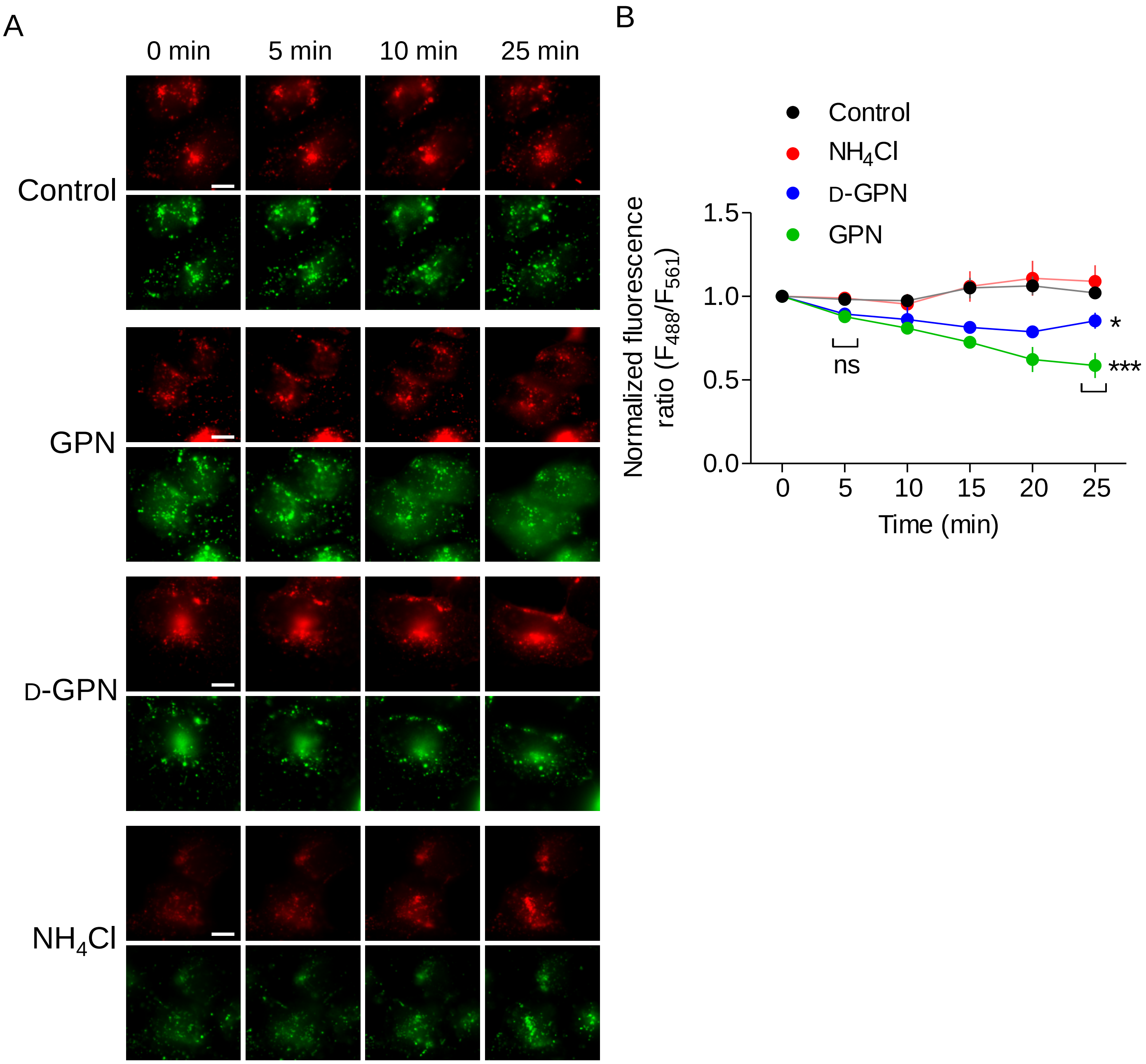

Fig. S3. Sustained exposure to GPN causes some loss of high-M_r fluorophores from lysosomes. (**A**) Typical wide-field images of HEK cells expressing LAMP1-mCherry (red) and with their lysosomes loaded with dextran-conjugated Alexa Fluor-488 (~10 kDa, green) and then treated with GPN (200 µM), D-GPN (200 µM), NH_4Cl (20 mM) or vehicle for the indicated times. Scale bars = 10 µm. Images were collected immediately before the additions and at 5-min intervals thereafter to minimize photobleaching. (**B**) Fluorescence ratios (F_{488}/F_{561}; i.e., $F_{Dextran}/F_{LAMP}$) were determined for each ROI enclosing a lysosome, and the ratios for each cell were normalized to the value immediately before treatment (t = 0). Results show mean ± SEM, n = 5 cells. $^{*}P < 0.05$, $^{***}P < 0.001$, ns, not significant, relative to time-matched control, 2-way ANOVA with Bonferonni test for the indicated comparisons only.

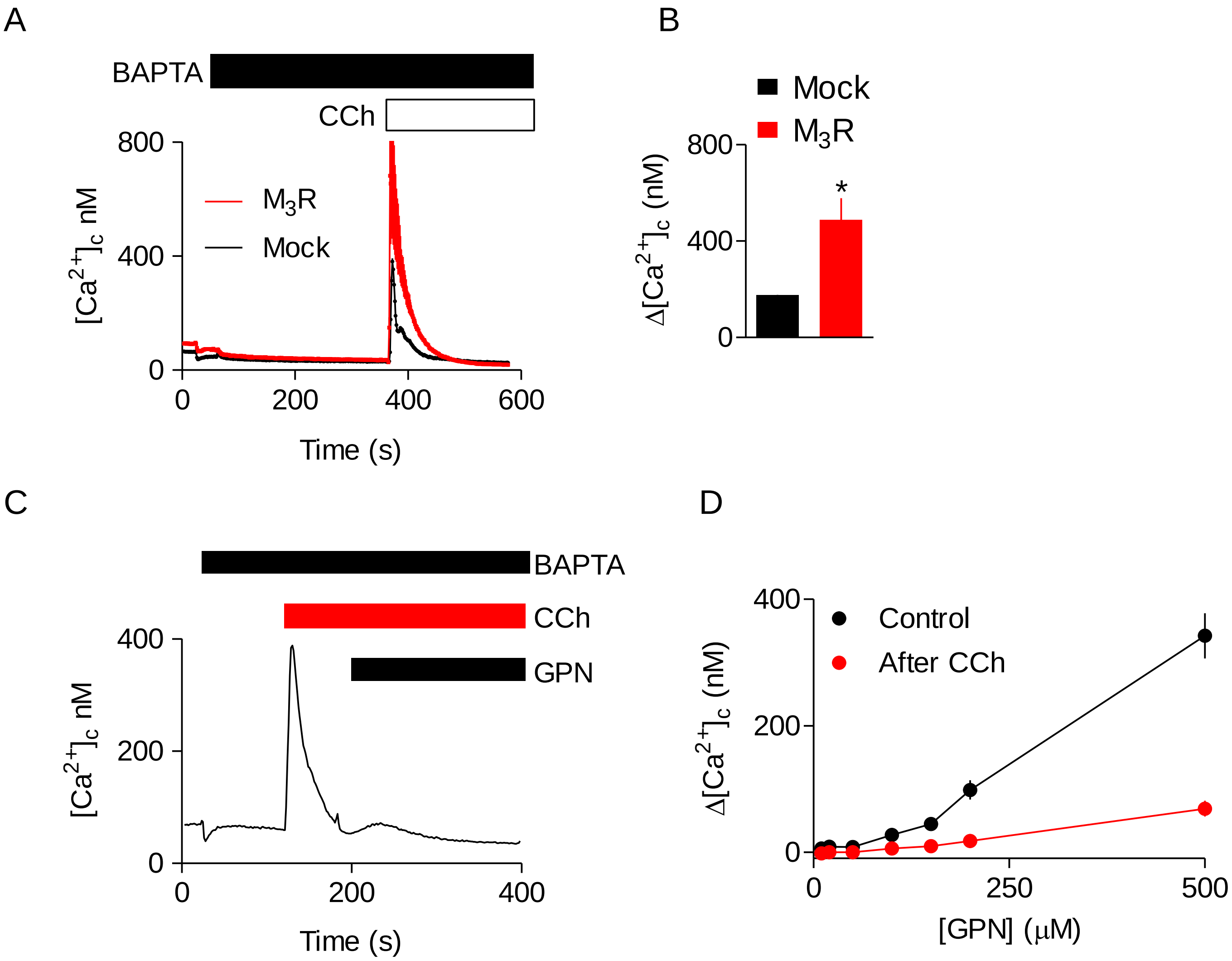

Fig. S4. Depleting ER Ca^{2+} stores by activating IP_3Rs rapidly attenuates GPN-evoked increases in $[Ca^{2+}]_c$. (**A**) HEK cells express M_3 muscarinic receptors (M_3Rs). When activated by carbachol (CCh), M_3Rs stimulate phospholipase C and thereby Ca^{2+} release from ER through IP_3Rs. However, endogenous expression of M_3 receptors is insufficient to fully deplete the ER of Ca^{2+} (Konieczny et al., 2017). We therefore used a viral vector, BacMam, to over-express M_3Rs in HEK cells. The results (mean ± SD from 2 replicates) show $[Ca^{2+}]_c$ recorded form a population of fluo 8-loaded HEK cells with or without (mock-infected) over-expressed M_3Rs and stimulated with a maximally effective concentration of CCh (1 mM). BAPTA (2.5 mM) was added before CCh to chelate extracellular Ca^{2+}. (**B**) Summary results (mean ± SEM, n = 3) show the peak increase in $[Ca^{2+}]_c$ ($\Delta[Ca^{2+}]_c$). $^*P$ < 0.05, Student's t-test. Responses to CCh were abolished in cells pre-treated with thapsigargin (1 μM, 15 min) to inhibit SERCA (results not shown). (**C**) HEK cells over-expressing M_3Rs were stimulated with CCh (1 mM) in Ca^{2+}-free HBS and then with GPN (200 μM). Results show mean of 3 replicates. (D) Summary results (mean ± SEM, n = 3) show responses to the indicated concentrations of GPN alone or after stimulation with CCh. The results demonstrate that depleting the ER of Ca^{2+} by stimulating IP_3Rs rapidly attenuates the increase in $[Ca^{2+}]_c$ evoked by GPN.

Journal of Cell Science • Supplementary information

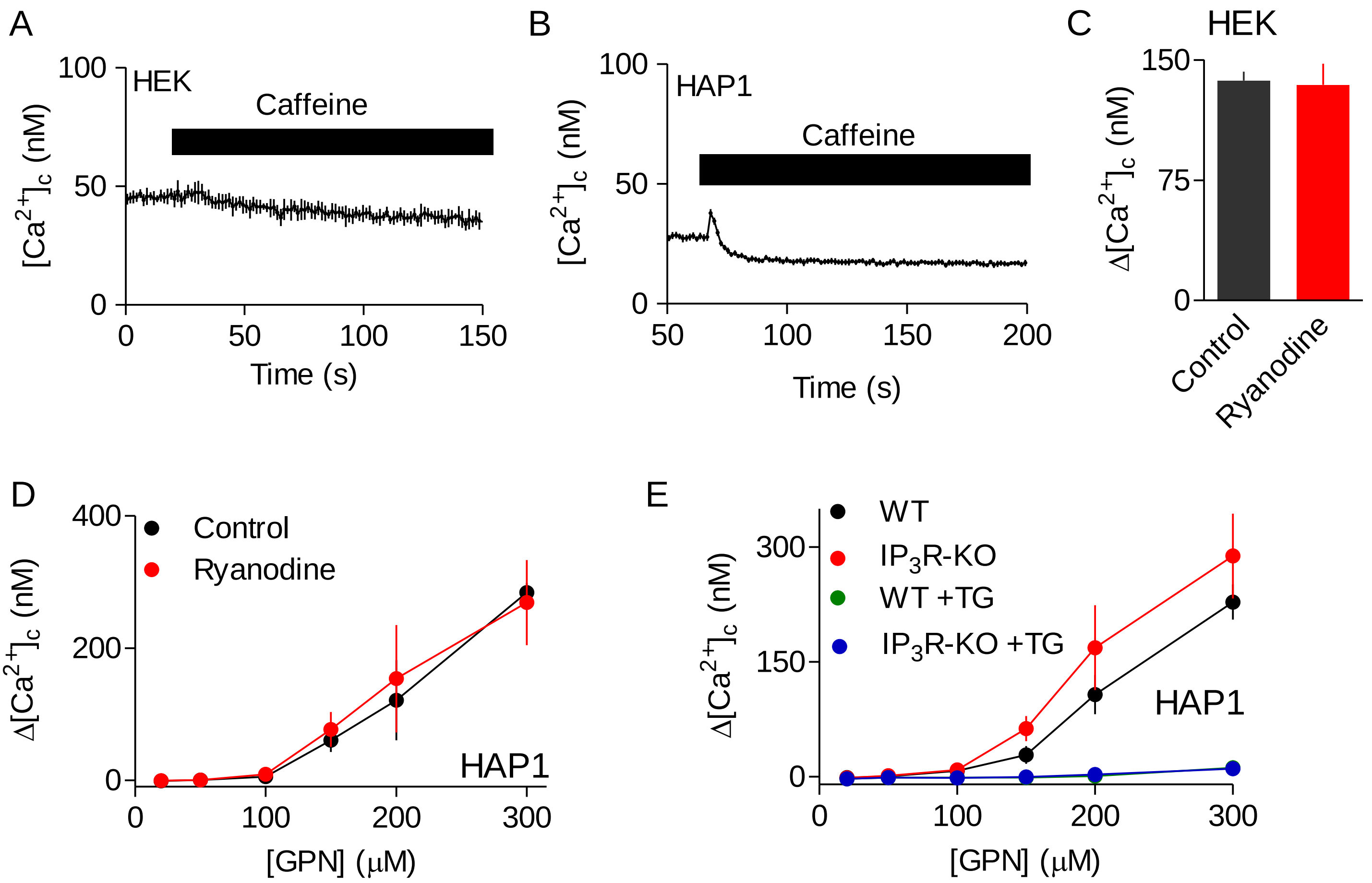

Fig. S5. Neither RyRs nor IP_3Rs contribute to cytosolic Ca^{2+} signals evoked by GPN. (**A,B**) Effects of caffeine (1 mM) in Ca^{2+}-free HBS on $[Ca^{2+}]_c$ in HEK (A) and HAP1 (B) cells. Traces show mean ± SD from 3 replicates. Similar results were obtained in 3 independent analyses. (**C,D**) Effects of GPN in Ca^{2+}-free HBS on the peak increase in $[Ca^{2+}]_c$ ($\Delta[Ca^{2+}]_c$) alone or after treatment with ryanodine (20 µM, 15 min) to inhibit RyRs. Results (mean ± SEM, *n* = 3, each with 3 replicates) are from HEK cells stimulated with 200 µM GPN (C) and HAP1 cells stimulated with the indicated concentrations of GPN (D). (E) Effects of GPN (200 µM) in Ca^{2+}-free HBS on HAP cells with (wild-type, WT) or without IP_3Rs (IP_3R-KO), alone or after treatment with thapsigargin (TG, 1 µM, 15 min). Results show mean ± SEM, *n* = 3, each with 3 replicates.

Journal of Cell Science • Supplementary information

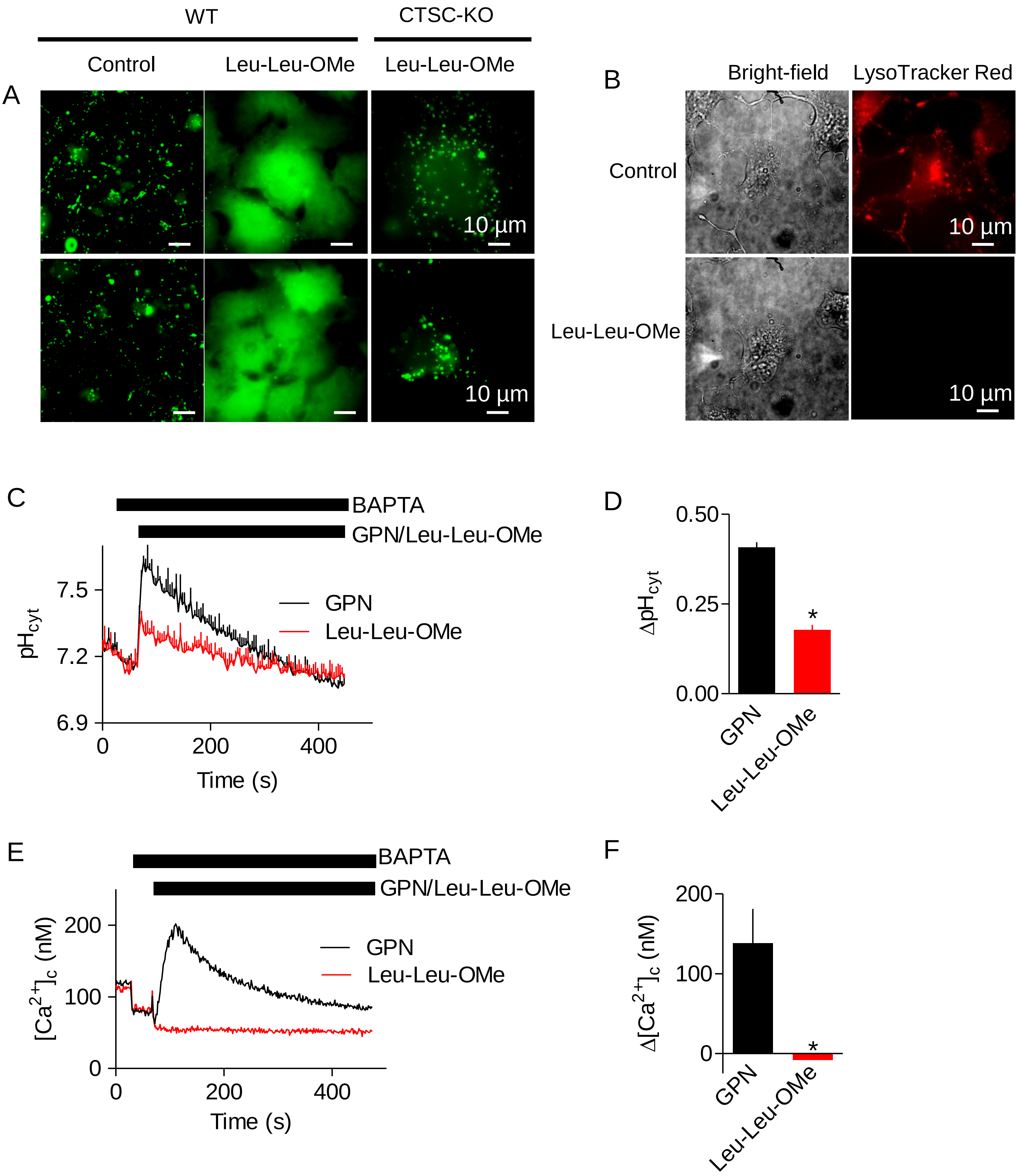

Journal of Cell Science • Supplementary information

Fig. S6. Leu-Leu-OMe ruptures lysosomes in a cathepsin C-dependent manner, but it does not increase $[Ca^{2+}]_c$. Figure on preceding page. (**A**) Two representative wide-field fluorescence images show the punctate distribution of endocytosed Alexa Fluor 488-dextran (M_r ~3,000) in HEK WT cells. Treatment with Leu-Leu-OMe (1 mM, 1 h) causes loss of the puncta in WT cells, but not in CTSC-KO cells. Results are typical of 3 experiments. Scale bar = 10 µm in all images. (**B**) Bright-field and wide-field fluorescence images of HEK cells loaded with LysoTracker Red (100 nM, 20 min) with or without Leu-Leu-OMe (1 mM, 1 h). Images are typical of three experiments. (**C**) Effects of GPN (200 µM) or Leu-Leu-OMe (1 mM) on pH_{cyt} in HEK cells. Results are mean ± SD for 3 replicates. (**D**) Summary results (mean ± SEM, *n* = 4, each with 3 replicates) show peak ΔpH_{cyt}. $^*P < 0.05$, Student's *t*-test. (**E**) BAPTA (2.5 mM) was added to chelate extracellular Ca^{2+}, before addition of GPN (200 µM) or Leu-Leu-OMe (1 mM) to fluo 8-loaded HEK cells. Results show mean for 3 replicates. (**F**) Summary results (mean ± SEM, *n* = 4-6, each with 3 replicates) show peak $\Delta[Ca^{2+}]_c$ evoked by GPN or Leu-Leu-OMe. $^*P < 0.05$, Student's *t*-test.

Journal of Cell Science • Supplementary information

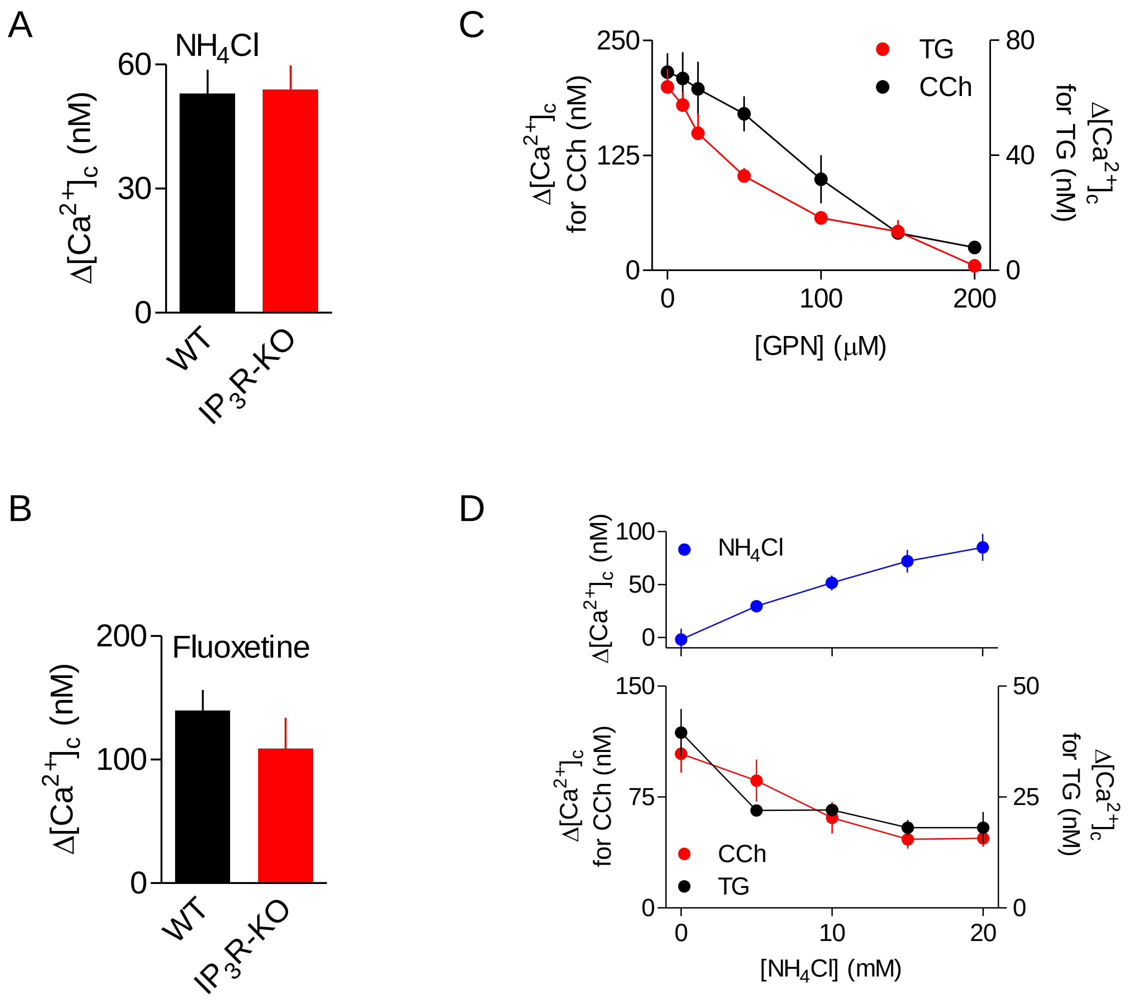

Fig. S7. Weak bases deplete the ER of Ca^{2+} independent of IP_3Rs. (**A,B**) Effects of NH_4Cl (20 mM) (A) or fluoxetine (300 µM) (B) in Ca^{2+}-free HBS on HEK cells with (WT) or without IP_3Rs (IP_3R-KO). Results show mean ± SEM, n = 6 (A) or n = 5 (B), each with 3 replicates. P = 0.90 (A) and P = 0.34 (B), Student's t-test. (**C**) HEK cells were stimulated with the indicated concentrations of GPN (15 min) in Ca^{2+}-free HBS before addition of carbachol (CCh, 1 mM) to stimulate IP_3 formation or thapsigargin (TG, 1 µM) to estimate the amount of Ca^{2+} remaining within the ER. Since CCh stimulates Ca^{2+} release, while TG, by inhibiting SERCA, unmasks a basal leak, the peak amplitudes of the Ca^{2+} signals are different for the two stimuli. Both, however, reveal a similar concentration-dependent decrease in ER Ca^{2+} content after GPN treatment. Results (mean ± SEM, n = 3, each with 3 replicates) show $\Delta[Ca^{2+}]_c$ for CCh or TG. (**D**) Similar analyses (mean ± SEM, n = 4-6, each with 3 replicates) show the effects of pre-treatment with NH_4Cl (15 min) on the subsequent $\Delta[Ca^{2+}]_c$ evoked by CCh or TG. The upper panel shows $\Delta[Ca^{2+}]_c$ evoked by NH_4Cl.

Journal of Cell Science • Supplementary information
